# A Survey Assessing Nonalcoholic Fatty Liver Disease Knowledge Among Hepatologists and Non‐Hepatologists in China

**DOI:** 10.1002/jgh3.70054

**Published:** 2024-12-10

**Authors:** Laura Colombo

**Affiliations:** ^1^ Department of Medical Affairs Viatris Milan Italy

**Keywords:** China, continuing medical education, hepatologists, nonalcoholic fatty liver disease, non‐hepatologists

## Abstract

**Background and Aim:**

A global increase in nonalcoholic fatty liver disease (NAFLD) prevalence has been observed in the last decade. This study assesses knowledge, awareness, and clinical practice gaps of hepatologists and non‐hepatologists in NAFLD management across hospitals in China.

**Methods:**

A web‐based quantitative survey was conducted, and participants included hepatologists (gastroenterologists and infectious disease specialists) and non‐hepatologists (internal medicine specialists, cardiologists, and pharmacists) from various hospitals across China.

**Results:**

In total, 1627 healthcare practitioners (HCPs) responded to the survey. This included 658 hepatologists and 969 non‐hepatologists. In comparison to 92.6% hepatologists, only 58.0% of non‐hepatologists were aware of NAFLD. A higher proportion of hepatologists (82.8%) performed screening for NAFLD compared to non‐hepatologists (56.9%). Majority of the hepatologists (70%) and non‐hepatologists (67%) were aware of the four primary recommendations for managing NAFLD. Only 11% of hepatologists did not manage NAFLD patients, mainly because they felt they did not have enough time (66.7%). Of the 36% non‐hepatologists who did not manage NAFLD, 78.4% stated that NAFLD is not their specialty, and 38.6% were not familiar with the treatment options.

**Conclusion:**

Most hepatologists were aware of and agreed to performing screening for NAFLD compared to non‐hepatologists. Both hepatologists and non‐hepatologists exhibited similar level of understanding on NAFLD management. However, a small percentage of both hepatologists and non‐hepatologists admitted that they did not manage NAFLD patients because they were not familiar with available treatment options. This underscores the importance of further educating HCPs involved in managing NAFLD.

## 1 Introduction

Nonalcoholic fatty liver disease (NAFLD) is among the leading causes of liver disease on a global scale. Around 2 million deaths annually and 4% of all deaths are due to liver disease. Globally, the incidence of NAFLD is 47 cases per 1000 population, with a higher rate observed in males than females [1]. The highest prevalence rate is observed in the Middle East and South America and the lowest in Africa [2]. According to a recent meta‐analysis, the combined regional prevalence of NAFLD in Asia was estimated to be 46.9 cases per 1000 person‐years (95% confidence interval [CI], 28.31–96.77) [3]. The burden of NAFLD/nonalcoholic steatohepatitis (NASH) has grown in China, yet little attention has been paid to this condition until recently. In 2019, China had 293.42 million NAFLD cases with a prevalence rate of 20.63 per 1000 people. The highest prevalence of NAFLD was highest in North China, and lowest in Southwest China [4].

NAFLD encompasses a spectrum that includes simple steatosis, advanced fibrosis, and, on rare occasions, progression toward cirrhosis. Cirrhosis has the potential to progress into hepatocellular carcinoma (2%–3% annually); however, NASH can also directly progress into hepatocellular carcinoma, even in the absence of cirrhosis [5, 6]. Patients diagnosed with simple steatosis consistently always have minimal risk of disease progression. Consequently, the timely identification of NAFLD is of utmost importance in order to implement effective therapeutic measures [7].

NAFLD is very common in people with type 2 diabetes mellitus (T2DM) and is also associated with a high risk of serious extrahepatic diseases, such as cardiovascular disease (CVD), and metabolic diseases such as obesity and dyslipidemia [8, 9, 10]. Furthermore, obesity is the most prevalent and extensively documented risk factor for NAFLD [11]. The European Association for the Study of the Liver (EASL), the European Association for the Study of Diabetes, and the European Association for the Study of Obesity recommend people with T2DM be screened for NAFLD [12]. Another risk factor for NAFLD is dyslipidemia with high serum triglyceride (TG) levels and low serum high‐density lipoprotein cholesterol (HDL‐C) levels [13].

NAFLD being a multisystem disease requires the approach of different specialties for diagnosis and treatment. A worldwide survey revealed a significant lack of awareness, knowledge, and management of NAFLD among doctors treating patients with heart and metabolic conditions. This trend was consistent across different specialties, with gastroenterologists and hepatologists being the most knowledgeable, followed by internists and general practitioners, and cardiologists being the least informed [14].

The majority of patients diagnosed with NAFLD are typically evaluated by medical professionals who are not specialized in gastroenterology or hepatology to diagnose NAFLD. Lack of awareness of NAFLD in non‐hepatologists is one of the reasons for missed or delayed diagnosis [15]. Recent findings show that > 25% of patients seeking specialist consultation already exhibit advanced fibrosis, thus highlighting the need for further education and training of nonspecialists aimed at promoting timely referral to specialists [16]. Adopting a multidisciplinary approach, with the involvement of clinicians specializing in gastroenterology, internal medicine, infectious diseases, and cardiology is strongly recommended for early intervention and effective management of NAFLD [17].

Considering the evolving landscape of NAFLD/metabolic‐associated fatty liver disease (MAFLD) definition, the growing projections of the disease in China, the multiple risk factors, and the different specialists that are involved, it is crucial to have all the stakeholders properly informed. The current study aims at determining the level of knowledge about current diagnostic and treatment patterns of NAFLD management among hepatologists and non‐hepatologists in China. This study also assesses the educational needs that could eliminate the barriers and gaps in the management of NAFLD.

## 2 Methods

### 2.1 Study Design

A web‐based quantitative survey on NAFLD knowledge and awareness was conducted with 1627 healthcare practitioners (HCPs), including hepatologists (gastroenterologists and infectious disease specialists) and non‐hepatologists (specialists in internal medicine, cardiology, and pharmacy) with approximately 300 HCPs in each specialty. The data were collected between May and June 2022 and was limited to the principal cities of China. HCPs were contacted through a third‐party vendor and the surveys were collected and analyzed by the vendor. The sponsor of the survey received the aggregated results.

The survey used multiple‐choice questions (some questions allowed selection of more than one choice). About 33 questions were included across five sections of the survey. The first section of the questionnaire included demographic variables, for example, name, age, gender, affiliations, specialties, and seniority. The second section consisted of questions concerning epidemiology, risk factors, and complications/comorbidities. The third section had questions related to screening and diagnostic methods. The fourth section focused on NAFLD management including dietary and lifestyle modification, available pharmacological treatments, and the primary ways to stay informed on NAFLD including current guidelines [18], literature, and online or in‐person medical education. The fifth section aimed at understanding the main barriers in the management of NAFLD in the opinion of the HCPs in China.

### 2.2 Data Analysis

The survey responses were analyzed, and categorical data were presented as numbers and percentages (*N* [%]). We were compliant with the local regulation of data privacy. For some of the questions, the level of knowledge was assessed with respect to current Chinese guidelines [18].

### 2.3 Ethics Approval

The study was conducted in line with the market research definition and in accordance with the European Pharmaceutical Market Research Association (EphMRA) [19] and British Healthcare Business Intelligence Association (BHBIA) [20] market research code of conducts. Its only purpose was to capture the opinion of the participants, and no clinical parameter, efficacy, or safety endpoints related to any treatment were investigated. Hence, in line with the guidance provided by the EphMRA, BHBIA, and the National Health Service Health Research Authority, the research does not qualify as clinical study and Research Ethics Committee review and approval was not required. All methods were carried out following the relevant guidelines and regulations.

Participants' consent was collected before participation in the web‐based survey.

## 3 Results

### 3.1 Demographic Characteristics of the Respondents

Section 1 of the questionnaire included questions on the demographic characteristics of the participating HCPs. In total, 1627 respondents (men: 972 [59.7%]; women: 655 [40.2%]) completed the web‐based quantitative survey. The respondents included doctors from public hospitals (nonuniversity affiliated), public universities, and private hospitals, pharmacists, nurses, and other professionals. The majority (82%) of the respondents were aged between 31 and 50 years. Overall, 76.1% of the HCPs work in tertiary hospitals, and the majority of the physicians had 11–20 years of experience. The survey respondents, based on their primary specialty, comprised gastroenterologists (*n* = 342), internal medicine doctors (*n* = 323), infectious disease specialists (*n* = 316), cardiologists (*n* = 320), and pharmacists (*n* = 326). For ease of comparing the level of knowledge and awareness on NAFLD, the respondents were grouped as hepatologists (gastroenterologists and infectious disease/tropical disease specialists; *n* = 658) and non‐hepatologists (specialists in internal medicine, cardiology, and pharmacy; *n* = 969) (Table 1).

**TABLE 1:** Demographics of participating physicians.

	Hepatologists, *N* (%)	Non‐hepatologists, *N* (%)	Total, *N* (%)
*N*	658	969	1627
Age			
≤ 30	6 (0.9)	35 (3.6)	41 (2.5)
31–40	290 (44.1)	447 (46.1)	737 (45.3)
41–50	258 (39.2)	339 (35.0)	597 (36.6)
51–60	92 (14.0)	130 (13.4)	222 (13.6)
> 60	12 (1.8)	18 (1.9)	30 (1.8)
Sex			
Male	423 (64.3)	549 (56.7)	972 (59.7)
Female	235 (35.7)	420 (43.3)	655 (40.2)
Practice setting			
Public hospital medical doctor (non‐university affiliated hospital)	350 (53.2)	381 (39.3)	731 (44.9)
Public university hospital medical doctor	282 (42.9)	222 (22.9)	504 (31.0)
Private hospital medical doctor	18 (2.7)	49 (5.1)	67 (4.1)
Pharmacist	7 (1.1)	311 (32.1)	318 (19.5)
Nurse	0 (0)	1 (0.1)	1 (0.1)
Other	1 (0.2)	5 (0.5)	6 (0.4)
Hospital type			
Primary hospital	13 (2.0)	97 (10.0)	110 (6.8)
Secondary hospital	96 (14.6)	182 (18.8)	278 (17.1)
Tertiary hospital	549 (83.4)	690 (71.2)	1239 (76.2)
Years in practice			
> 5 years	8 (1.2)	44 (4.5)	52 (3.2)
5–10 years	120 (18.2)	189 (19.5)	309 (19.0)
11–20 years	342 (52.0)	432 (44.6)	774 (47.6)
21–30 years	135 (20.5)	216 (22.3)	351 (21.5)
> 30 years	53 (8.1)	88 (9.1)	141 (8.7)
Primary specialty			
*N*	658	969	1627
Gastroenterology	342	0	342 (21.0)
Pharmacy	0	326	326 (20.0)
Internal medicine	0	323	323 (19.8)
Cardiology	0	320	320 (19.6)
Infectious disease	316	0	316 (19.4)

Abbreviation: *N*, total number of the participants.

### 3.2 Level of Knowledge of NAFLD Definition and Epidemiology

**FIGURE 1:**
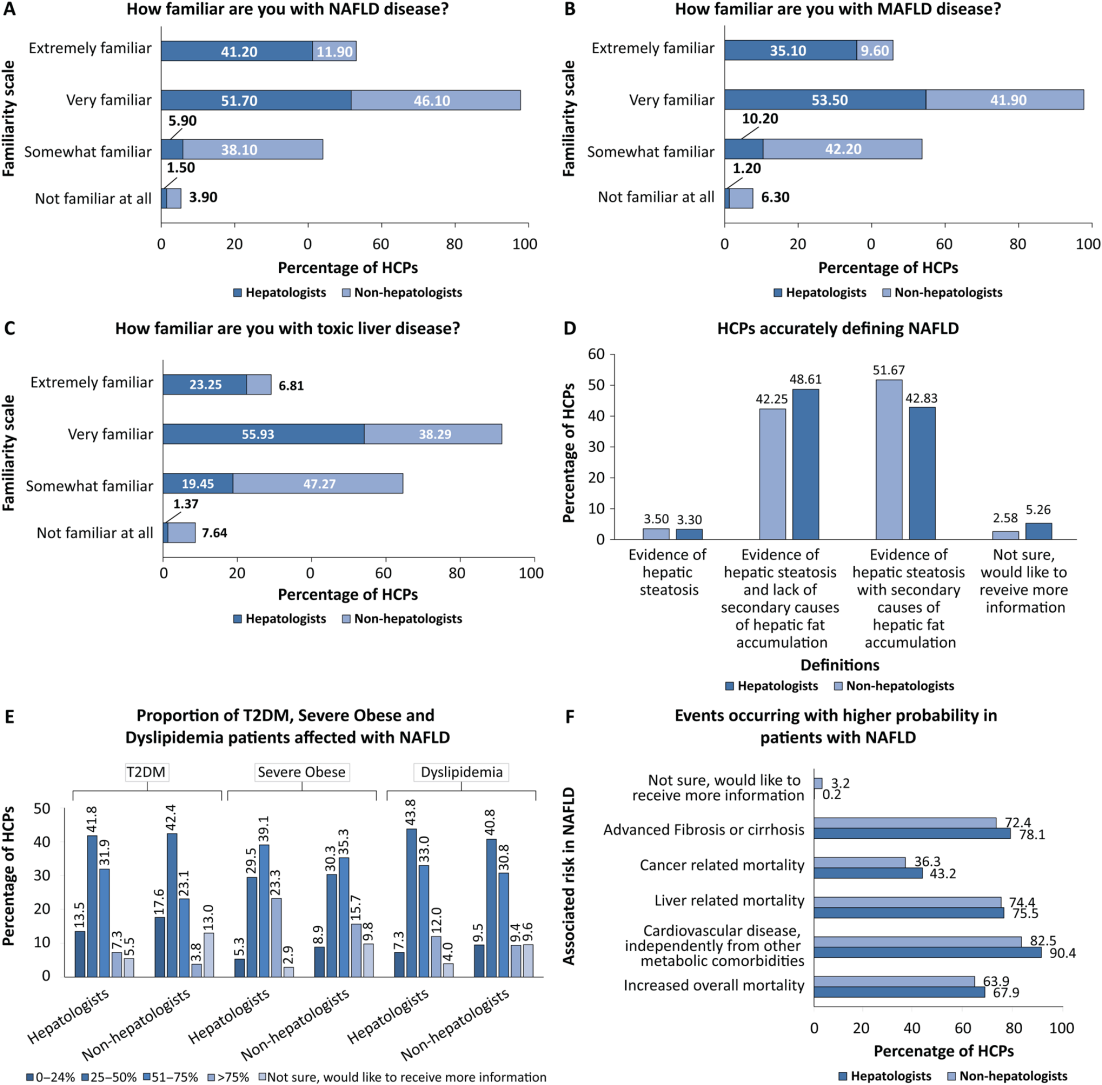
Knowledge about NAFLD, MAFLD, and toxic liver disease, definition, comorbid, and associated diseases among hepatologists and non‐hepatologists in China (A‐F). HCPs: healthcare professionals; MAFLD: metabolic dysfunction‐associated fatty liver disease; NAFLD: nonalcoholic fatty liver disease; T2DM: type 2 diabetes mellitus.

Section 2 of the questionnaire focused on the NAFLD definition and epidemiology. The majority of the hepatologists (overall: 92.6%; extremely familiar: 41.2%; very familiar: 51.7%) demonstrated a high level of familiarity with NAFLD. Conversely, among non‐hepatologists, a remarkably lower proportion of respondents (overall 58%; extremely familiar: 11.9%; very familiar: 46.1%) exhibited familiarity with NAFLD (Figure 1A). Similarly, a higher proportion of hepatologists were either extremely or very familiar with MAFLD (88.6%) and toxic liver disease (79.2%) in comparison to non‐hepatologists (MAFLD: 51.5%; toxic liver disease: 45.1%). More non‐hepatologists expressed no familiarity with MAFLD (6.3%) and toxic liver disease (7.6%) compared to hepatologists (1.2% and 1.4%, respectively) (Figure 1B,C).

In response to the survey question on the most accurate definition of NAFLD, less than 50% of both hepatologists (42.2%) and non‐hepatologists (48.6%) agreed with the occurrence of hepatic steatosis (HS) and absence of secondary causes of hepatic fat accumulation, which is in accordance with international clinical guidelines. The majority of hepatologists (51.7%) defined NAFLD as HS with a secondary cause of hepatic fat accumulation, which is not the most appropriate definition of NAFLD (Figure 1D).

Another set of survey question was aimed at assessing the understanding of hepatologists and non‐hepatologists regarding the prevalence of NAFLD in the general population as well as specific patient populations (i.e., patients with T2DM, severe obesity, and dyslipidemia) in China. A comparatively higher percentage of hepatologists correctly estimated the proportion of the general population in China that may be affected by NAFLD (hepatologists: 72.2%; non‐hepatologists: 60.7%). In addition, a higher proportion of hepatologists compared to non‐hepatologists were aware of NAFLD prevalence in the various patient populations in China (T2DM—hepatologists: 73.7%; non‐hepatologists: 65.5%; severe obesity—hepatologists: 62.3%; non‐hepatologist: 51%; dyslipidemia—hepatologists: 88.8%; non‐hepatologists: 80.9%). Further, a higher proportion of non‐hepatologists were not sure about the prevalence of NAFLD in these patient populations in China compared to the hepatologists (Figure 1E). The majority of both hepatologists (78.3%) and non‐hepatologists (77.6%) were aware that NAFLD is more prevalent among men than women in China. Both hepatologists and non‐hepatologists demonstrated a similar awareness of the events that may occur with a higher probability in patients with NAFLD. However, hepatologists demonstrated a higher awareness of, and were more likely to identify, CVD (90.4% vs. 82.5%), advanced fibrosis or cirrhosis (78.1% vs. 72.4%), and cancer related mortality (43.2% vs. 36.3%) than non‐hepatologists as events most frequent in patients with NAFLD (Figure 1F). Despite knowing the potential events that may arise in patients with NAFLD, only 35% of hepatologists and 27% of non‐hepatologists accurately identified all five of the most common events that occur in NAFLD patients, as outlined in the guidelines.

### 3.3 Screening and Diagnosis of NAFLD

**FIGURE 2:**
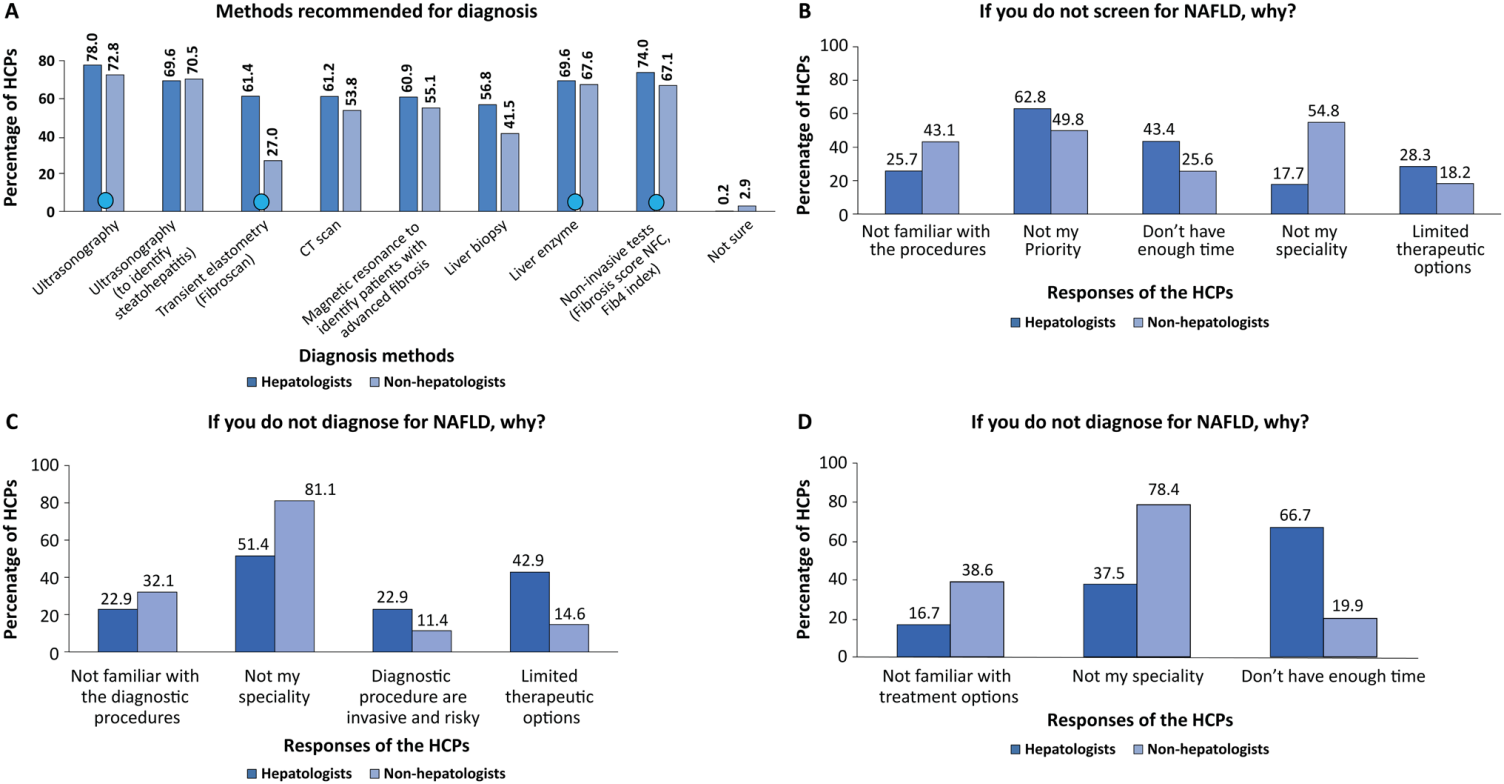
Screening, diagnosing, and managing of NAFLD by HCPs and recommended diagnostic methods (A‐D). 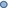 indicates correct diagnostic options. CT: computed tomography; HCPs: healthcare professionals; NAFLD: nonalcoholic fatty liver disease.

In Section 3 of the questionnaire, the HCPs answered to questions aimed to understand whether they screened, diagnosed, and managed NAFLD patients. Table 2 summarizes the responses received from hepatologists and non‐hepatologists. A comparatively higher proportion of hepatologists (82.8%) confirm to perform screening for NAFLD compared to non‐hepatologists (56.9%). Among the hepatologists who do not screen for NAFLD, the majority selected the reason as “not my priority,” while the majority of the non‐hepatologists not performing screening for NAFLD provided the reason of “not my specialty” (Figure 2B). Overall, 94.7% of hepatologists agreed to be performing a diagnosis of NAFLD, compared to only 59.1% of the non‐hepatologists (Table 2). Among those who responded negative (hepatologists: 5.3%; non‐hepatologists: 40.9%) regarding diagnosing NAFLD, the majority of both hepatologists and non‐hepatologists provided the reason of “not my specialty,” though the proportion of non‐hepatologists with this response was much higher (81.1%) compared to hepatologists (51.4%). Further, a notable proportion of hepatologists (42.9%) selected “limited therapeutic options” as the reason for the same. When asked whether they managed patients with NAFLD, the majority of hepatologists (89.1%) and non‐hepatologists (64.2%) responded in the affirmative (Figure 2C). Among those who replied in negative, most hepatologists selected the reason “I don't have enough time,” whereas most non‐hepatologists responded as “not my specialty.” In addition, a notable proportion of both hepatologists (16.7%) and non‐hepatologists (38.6%) stated that they were not familiar with treatment options for NAFLD (Figure 2D).

**TABLE 2:** Diagnosing, screening, and managing of NAFLD patients.

	Do you screen for NAFLD?		Do you diagnose NAFLD?		Do you manage NAFLD patients?	
	Hepatologists	Non‐hepatologists	Hepatologists	Non‐hepatologists	Hepatologists	Non‐hepatologists
Total	658	969	658	969	658	969
Yes (%)	82.8	56.9	94.7	59.1	89.1	64.2
No (%)	17.2	43.1	5.3	40.9	10.9	35.8

Abbreviation: NAFLD: nonalcoholic fatty liver disease.

Irrespective of whether they are involved in screening or diagnosis of NAFLD, the HCPs were asked to select from a list of seven items the ones that may, in their opinion, suggest a need for NAFLD screening. Despite the similarity in the pattern of awareness regarding the items that may indicate the necessity for NAFLD screening among different specialty groups, hepatologists exhibit a higher likelihood of accurately identifying these items compared to non‐hepatologists (Table 3). Only 25% of hepatologists and 17% of non‐hepatologists were able to correctly identify all the seven items (one or more metabolic risk factors, liver steatosis, abnormal liver chemistry, NAFLD familiarity, hypothyroidism, polycystic ovary syndrome, and sleep apnea) that call for NAFLD screening (Table 3).

**TABLE 3:** Understanding the need to screen for NAFLD.

Question: Even if you don't always screen or diagnose NAFLD, please select all the items below that may suggest need of screening for NAFLD		
	Hepatologists (%)	Non‐hepatologists (%)
One or more metabolic risk factors, such as diabetes or insulin resistance, hypertension, dyslipidemia, obesity	95.3	90.7
Liver steatosis	91.9	85.9
Abnormal liver chemistry	91.0	85.1
NAFLD familiarity	86.6	83.8
Hypothyroidism	42.4	34.6
Polycystic ovary syndrome	41.3	29.1
Sleep apnea	42.1	33.3
Not sure, would like to receive more information	—	3.0

Abbreviation: NAFLD: nonalcoholic fatty liver disease.

When asked about the methods recommended for the diagnosis of NAFLD, 35% of the hepatologists correctly identified the four recommended methods for diagnosing NAFLD (namely, ultrasonography, noninvasive tests such as the fibrosis score NFS and Fib‐4 index, liver enzyme analysis, and transient elastometry [FibroScan]) compared to 15% of non‐hepatologists. Noteworthy, only 27% of non‐hepatologists were aware of the importance of FibroScan as a recommended noninvasive diagnosis method compared to 61.4% of hepatologists (Figure 2A).

The HCPs were also questioned regarding their insights on the indicators of liver biopsy. Hepatologists demonstrated a better understanding of the factors suggesting a liver biopsy compared to the non‐hepatologists, and 22% of the hepatologists correctly identified the four relevant factors, which include the risk of steatohepatitis and/or cirrhosis, other conditions causing steatohepatitis that cannot be excluded, presence of metabolic syndrome, and high NAFLD fibrosis score (or similar), compared to only 14% of non‐hepatologists (Figure S1).

### 3.4 Management of NAFLD Patients

In this study, the Section 4 of the questionnaire assessed the level of understanding regarding the management of NAFLD among HCPs in China. Majority of the respondents, both hepatologists (70%) and non‐hepatologists (67%) were aware of the four primary recommendations for managing NAFLD, which include lifestyle modifications, dietary modification, pharmacological treatment, and bariatric surgery; however, relatively fewer considered bariatric surgery (Table S1). Around 81% of hepatologists and 77% of non‐hepatologists correctly identified the three main dietary recommendations for NAFLD management, which include low‐lipid, low‐calories, and low carbohydrate diet. In response to the question related to lifestyle modifications, only 5% of both hepatologists and non‐hepatologists identified all the necessary lifestyle modifications, which include avoiding hepatotoxic drugs, avoiding alcohol, weight loss of 10%, and weight loss of 3%–5%, highlighting a need for improved awareness of both the specialists and nonspecialists in this regard (Table S1).

**FIGURE 3:**
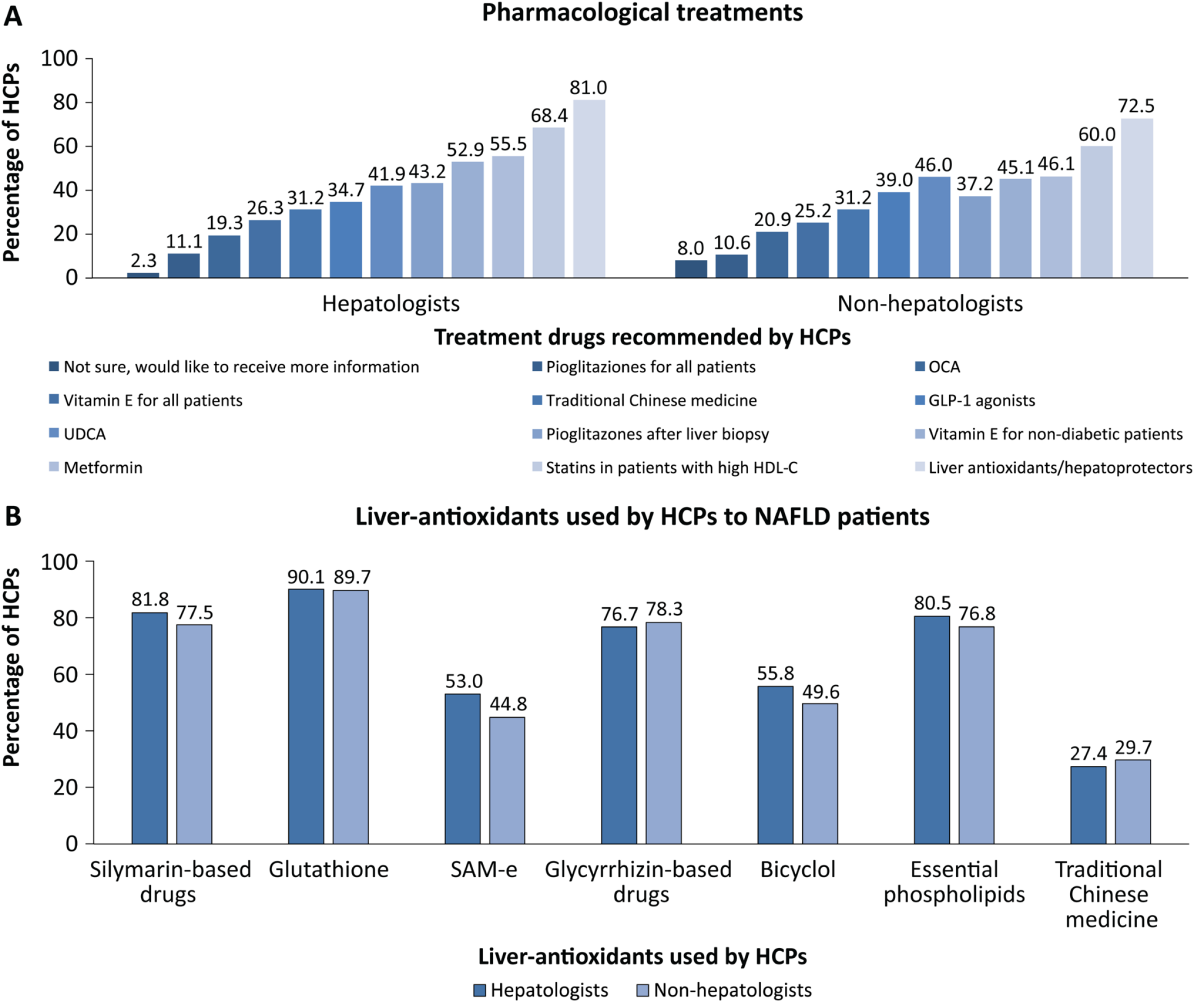
Pharmacological and liver‐antioxidant therapies adopted by HCPs to treat NAFLD patients (A‐B). GLP‐1: glucagon‐like peptide 1; HCPs: healthcare professionals; HDL‐C: high‐density lipoprotein cholesterol; NAFLD: nonalcoholic fatty liver disease; OCA: obeticholic acid; SAM‐e: S‐adenosylmethionine; UDCA: ursodeoxycholic acid.

**FIGURE 4:**
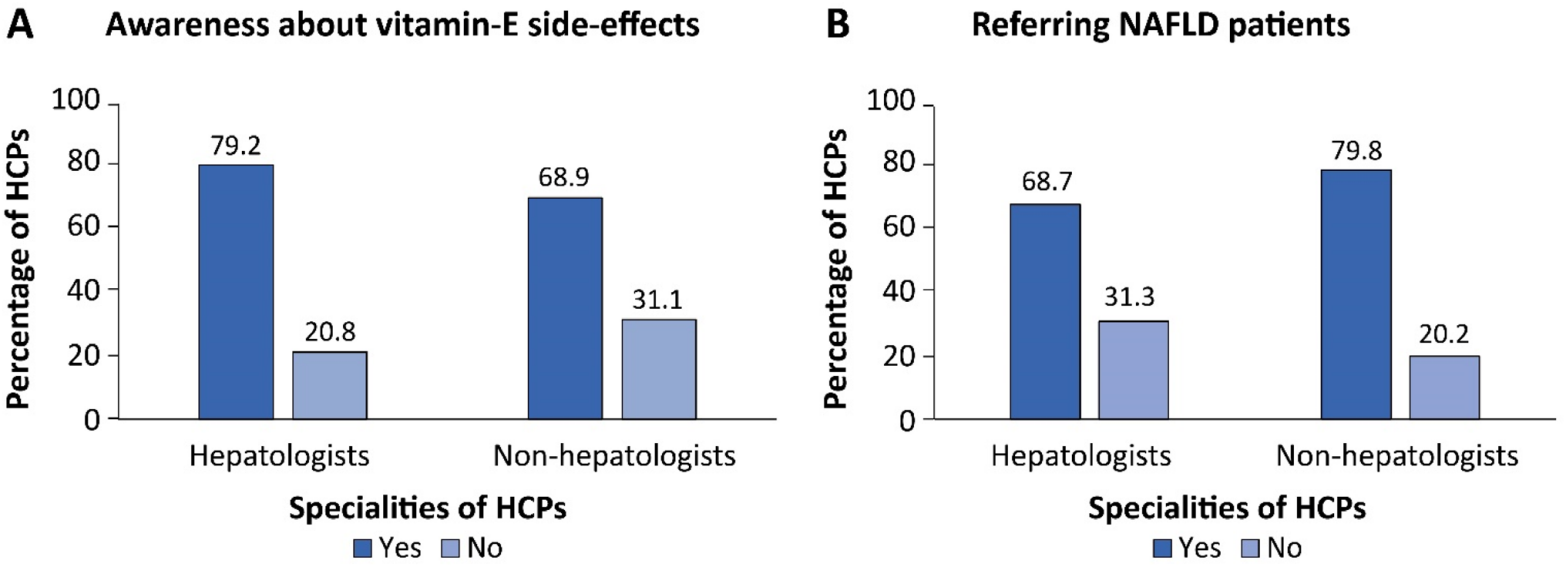
Awareness of vitamin E side effects among HCPs (A) and referring of NAFLD patients to gastroenterologists/hepatologists (B). HCPs: healthcare professionals; NAFLD: nonalcoholic fatty liver disease.

The HCPs were asked to select the options that they currently adopt from a list of available pharmacological treatment options for NAFLD. The treatment options enlisted included vitamin E to all patients, vitamin E to nondiabetic patients, pioglitaziones to all patients, pioglitaziones after liver biopsy, metformin, ursodeoxycholic acid, obeticholic acid, statins in patients with increased level of HDL‐C, glucagon‐like peptide 1 agonist, liver antioxidants/hepatoprotectors, and traditional Chinese medicines. Notably, liver antioxidants, alone or in combination with other treatments, were the most chosen treatment by both hepatologists (81%) and non‐hepatologists (72.5%) (Figure 3A). Antioxidants, namely glutathione, silymarin, and phospholipids, were the top three drugs most frequently chosen by both hepatologists and non‐hepatologists, with > 77% of HCPs selecting them. However, glutathione emerged as the primary choice of liver antioxidant by HCPs (Figure 3B). Both hepatologists (68.4%) and non‐hepatologists (60.0%) stated that they adopt statins for patients with high HDL‐C. Other notable pharmacological treatment options selected by both hepatologists and non‐hepatologists included metformin and vitamin E. The use of vitamin E in NAFLD patients can result in side effects. The survey findings also indicated that a considerable percentage of both hepatologists (20.9%) and non‐hepatologists (31.1%) demonstrated a lack of information about the potential side effects linked with Vitamin E (Figure 4A,B).

In response to the question of whether the HCPs refer patients with NAFLD to gastroenterologists, the majority of both hepatologists (68.7%) and non‐hepatologists (79.8%) responded in affirmative.

Another survey question examined the knowledge of HCPs regarding drugs that may worsen NAFLD. The drugs identified as worsening NAFLD include statins, nonsteroidal anti‐inflammatory drugs (NSAIDs), paracetamol, azathiopirine, methotrexate (MTX), quinine, valproate, tamoxifen, corticosteroid, amiodarone, and certain antibiotics, herbal supplements, and food supplements. The findings of the survey indicate that a majority of the HCPs (55%) were not aware of the fact that certain drugs can worsen the condition of NAFLD. Majority of the hepatologists and non‐hepatologists selected paracetamol and MTX as the top two drugs that caused worsening of NAFLD (Table S2). Compared to non‐hepatologists, hepatologists were much more likely to be aware of which drugs may worsen NAFLD. However, a proportion of HCPs were not aware of which drugs may worsen the disease (Table S2).

When asked about the usual ways through which the HCPs stay informed about NAFLD, the majority of hepatologists (97.4%) and non‐hepatologists (86.1%) responded that they read guidelines and peer‐reviewed journals. Further, 89.5% hepatologists and 82.1% non‐hepatologists said that they attend online medical education. A slightly higher, though not significant, proportion of non‐hepatologists (18.7%) compared to hepatologists (13.1%) were of the opinion that they would like to be more informed (Table S3).

### 3.5 Understanding Barriers to Manage NAFLD Patients

Section 5 of this survey was aimed to understand the challenges in NAFLD management that are faced by HCPs in China. The survey response by both hepatologists and non‐hepatologists identified lack of compliance by patients and time constraints as the top two barriers in NAFLD management. In comparison to hepatologists (41.9%), a higher proportion of non‐hepatologists (51.1%) selected lack in medical education of doctors as a barrier to NAFLD management (Table S4). Furthermore, one in five HCPs found discussing NAFLD with patients uncomfortable (Table S4).

## 4 Discussion

NAFLD is a leading cause of liver disease globally [1]. To assess the knowledge and management approaches of NAFLD, we performed a comprehensive survey among 1627 physicians across China. This survey explores the knowledge and awareness of hepatologists and non‐hepatologists on NAFLD. The survey comprised questions that were divided into five sections namely: demographic characteristics of HCPs, epidemiology, screening and diagnosis, management, and barriers to managing NAFLD.

NAFLD has been extensively discussed in the guidelines of the Asian Pacific Association for the Study of the Liver (APASL) which covers the various aspects of MAFLD (formerly known as NAFLD) [21]. MAFLD is an overarching term that encompasses a broad range of clinicopathological findings. The diagnosis of MAFLD requires evidence of steatosis in > 5% of hepatocytes and the exclusion of known causes (i.e., alcohol consumption, use of steatogenic medication, or hereditary disorders) for secondary hepatic fat accumulation. To differentiate between MAFLD and NASH, a liver biopsy is the gold standard to assess the presence of steatohepatitis. However, this invasive procedure should be performed only in individuals with suspected advanced fibrosis or doubts about the etiology of the liver disease. Noninvasive tests, such as abdominal ultrasonography, transient elastography, and magnetic resonance imaging may also be useful to detect and quantify the amount of HS and fibrosis to diagnose MAFLD [22]. In the year 2023, an independent committee recommended the name metabolic dysfunction‐associated steatotic liver disease instead of NAFLD or MAFLD for the patients who meet one of the five parameters of metabolic dysfunction: (i) body mass index ≥ 23 kg/m^2^ or waist circumference ≥ 90 cm for men and ≥ 85 cm for women; (ii) fasting serum glucose ≥ 100 mg/dL or those with T2DM or undergoing T2DM treatment; (iii) blood pressure ≥ 130/85 mmHg or undergoing antihypertensive drug treatment; (iv) TGs ≥ 150 mg/dL or undergoing lipid lowering treatment; and (v) HDL‐C ≤ 40 mg/dL for men and ≤ 50 mg/dL for women or undergoing lipid lowering treatment, on the basis of the presence of HS [23, 24]. The Chinese guideline suggests that the definition for fatty liver disease is associated with comorbidities of metabolic dysfunction. This recommendation includes a change in the name from NAFLD to MAFLD and adoption of a set of positive criteria for disease diagnosis that are independent of alcohol intake or other liver diseases [25]. According to the American Association for the Study of Liver Diseases (AASLD) guideline, NAFLD is defined by the presence of (1) evidence of HS, either through imaging or histology, and (2) absence of secondary causes of hepatic fat accumulation such as significant alcohol consumption, long‐term use of a steatogenic medication, or monogenic hereditary disorders [9]. Additionally, the EASL guideline outlines NAFLD as the abnormal buildup of hepatic fat, which is linked to insulin resistance (IR). It is identified by the presence of steatosis in > 5% of hepatocytes through histological analysis or by a proton density fat fraction > 5.6% assessed by proton magnetic resonance spectroscopy (1HMRS) or quantitative fat/water selective magnetic resonance imaging [26].

Long‐term observational studies reveal that cardiovascular risk factors contribute majorly to mortality in patients with NAFLD [27]. According to the APASL guideline, MAFLD represents a component of a complex disease involving multiple systems, hence it is not surprising that CVD is one of its primary complication [21]. The Chinese guideline, also categorizes MAFLD in alignment with other metabolic disorders, including diabetes, chronic kidney disease, and CVD [25]. The AASLD guideline emphasizes the cardiovascular risks in patients with NAFLD and recommends screening for CVD, the leading cause of death that is not linked to other metabolic comorbidities [9]. A position paper in 2010 by the EASL, acknowledges CVD as an extraneous manifestation of NAFLD. The screening for cardiovascular risk factors is a distinct recommendation and is therefore regarded as a fundamental component in the management of a patient with NAFLD [27].

### 4.1 Level of Awareness About NAFLD: A Substantial Obstacle to Overcome

Despite being the primary cause of incidental liver test abnormalities and a burgeoning global health concern, NAFLD remains largely undervalued by HCPs who are not liver specialists. This includes primary care physicians, specialists from other medical fields, and physicians who provide care for individuals with metabolic disorders [28]. Studies across the world have indicated that a significant proportion of medical professionals lack sufficient knowledge about NAFLD, underestimate the frequency and risks associated with the condition, and are not well‐informed about its proper evaluation [29, 30]. In the survey conducted as part of this study, the majority of the hepatologists demonstrated a high level of familiarity with NAFLD, MAFLD, and toxic liver disease. However, a considerable number of hepatologists were unable to correctly identify the definition of NAFLD. Furthermore, both hepatologists and non‐hepatologists alike faced challenges in determining whether the inclusion or exclusion of hepatic fat accumulation accompanied by HS should be considered as a defining criterion for NAFLD.

### 4.2 Multi‐Stakeholder Management of NAFLD and the Contribution of Non‐Hepatologists

NAFLD encompasses a range of conditions related to metabolic syndrome including obesity, T2DM, dyslipidemia, and hypertension [9]. Therefore, it is crucial to adopt a comprehensive and collaborative approach involving multiple disciplines and stakeholders is necessary to ensure optimal patient care and effectively manage the accompanying comorbidities. In order to facilitate discussions on this matter, a global public health and NAFLD consensus was held in Barcelona in 2022. This consensus brought together approximately 100 stakeholders from diverse disciplines to exchange knowledge and experiences regarding healthcare strategies for NAFLD. The program provided a platform for experts such as primary care physicians, hepatologists, endocrinologists, nutritionists, public health experts, and nurses to discuss various models of care. The consensus emphasized the importance of evaluating the benefit to patients and cost‐effectiveness of multidisciplinary models of care based on noninvasive tests. Primary care physicians and endocrinologists are the first‐line providers who should consider adopting these models. It is recommended to utilize delivery models that overcome the time constraints of primary care physicians through use of automated methods of screening and risk stratification, multidisciplinary ‘metabolic’ clinics or training, virtual care, nurse coordinators across disciplines, web‐based applications for longitudinal care, and virtual assistants. Additionally, it is essential to create, provide, and monitor clear guidance endorsed by appropriate professional societies regarding preventive hepatology and specific criteria for referral to specialty care, including hepatology, cardiology, endocrinology, weight management experts, bariatric surgeons, and fitness experts. The consensus reached during the discussion recommended a two‐tiered approach to risk stratification for T2DM, metabolic syndrome, obesity/overweight, and abnormal aminotransferases incidental HS. The consensus also emphasized the sequential use of noninvasive tests to identify patients with advanced liver disease. The first step involves using the FIB‐4 score to rule out cases with advanced disease and the second step involves assessment via liver stiffness measurement and enhanced liver fibrosis. The success of clinical pathways relies on the awareness and actions of primary care and non‐hepatologists. They need to recognize risk factors, order liver enzyme tests, interpret results, and refer patients to specialists when necessary [31].

### 4.3 Awareness Among Chinese HCPs on Treatment Options for NAFLD

NAFLD is a prominent health issue, yet over 100 countries lack a comprehensive public health response. Therefore, there is an urgent need for policies and strategies to manage NAFLD on both, national and global levels are urgently needed [32]. As per the insights gathered from the responses of Chinese HCPs to survey questions in this study, knowledge on NAFLD was limited and differed among the medical specialties. The hepatologists demonstrated better awareness on NAFLD treatment and management compared with non‐hepatologists. Further, we evaluated the level of awareness among HCPs regarding the impact of diet and lifestyle NAFLD. The findings indicated that a majority of both hepatologists and non‐hepatologists possessed knowledge about the four main recommendations for managing NAFLD. These recommendations encompassed implementing dietary and lifestyle modifications, considering pharmacological treatment options, and potentially exploring bariatric surgery. Both groups of HCPs demonstrated awareness of these aforementioned recommendations. Furthermore, the hepatologists and non‐hepatologists accurately identified the necessary lifestyle modifications, such as achieving a weight loss of 10% and a weight loss range of 3%–5%. All HCPs were in agreement with respect to the occurrence of HS; however, there was a lack of clarity on whether the presence or absence of secondary causes of hepatic fat accumulation with HS should be considered to define NAFLD. Nonetheless, the cardiovascular implications were identified as the most significant adversities for individuals diagnosed with NAFLD by both specialists and nonspecialists.

A cross‐sectional survey shared insights on the medical experiences of patients with NASH and the involvement of hepatologists/gastroenterologists in diagnosing and treating NASH. The study emphasized the importance of raising awareness and improving the diagnosis rate to enhance treatment outcomes [33]. Consequently, it is imperative to prioritize the adoption of a healthy lifestyle and weight reduction, as these factors play a pivotal role in NAFLD management [9].

There are no pharmacologic agents specifically licensed for the treatment of NAFLD [34]. A study conducted on essential phospholipid extracted from soybean limits liver damage, restores membrane structure and fluidity, inhibits or corrects fibrotic processes, influences apoptosis, and modulates lipid metabolism and has also been shown to have anti‐inflammatory and antioxidant properties [35, 36]. The hepatologists who participated in the current study selected phospholipids, antioxidants, and silymarin, as the recommended drug therapy for NAFLD patients. Silymarin, derived from the milk of thistle plant, has demonstrated hepatoprotective properties in patients with NAFLD [37]. Further, a significant number of hepatologists chose a combination of liver antioxidants along with other drugs as the treatment option. It is worth noting that a considerable proportion of both hepatologists and non‐hepatologists were not aware of the side effects of Vitamin E, although most HCPs were aware of the side effects associated with drugs such as NSAIDs, statins, and other medications.

### 4.4 Medical Education Among HCPs

This survey‐based study identifies the gaps in the level of knowledge and overall awareness of both hepatologists and non‐hepatologists in China on different aspects of NAFLD management. The findings of this study show that a notable proportion of non‐hepatologists lacked in‐depth knowledge on NAFLD. When asked about the sources of information commonly used for acquiring information on NAFLD, the majority of the HCPs expressed that they referred to relevant guidelines and articles published in peer‐reviewed journals to keep themselves updated on new developments in the field. In addition, HCPs also agreed that they attended online medical education programs as a source of information on NAFLD.

It is noteworthy that well‐designed continuing medical education programs focusing on the role of non‐hepatologists, hepatologists, and other primary care team members in NAFLD management, which includes guidance on referral and comanagement with specialists, is the need of the hour for HCPs in China. Such focused programs that are designed using the latest international guidelines will help change the mindset of primary care providers regarding their responsibilities and clinical significance in diagnosis and early treatment of NAFLD [38].

### 4.5 Strengths and Limitations

The main strength of our study was the extensive participation and coverage of the HCPs (hepatologists and non‐hepatologists) from various regions across China, highlighting the robustness of the study. This helped us in better comparative assessment of knowledge, screening, and treatment practices between hepatologists and non‐hepatologists in the management of NAFLD.

The present study was limited by insufficient sample size, as the number of participants could have been larger to obtain a more representative view of all the doctors in China.

## 5 Conclusion

This study has highlighted the difficulties faced in screening, diagnosing, treating, and managing NAFLD, which are directly related to the educational needs of physicians responsible for the care of patients. It strongly emphasizes the necessity for a better understanding of NAFLD and proposes that continuous medical education for doctors on the disease condition is the most effective way to move forward. Despite the increasing burden of NAFLD, there still exists a substantial gap in knowledge regarding its identification, diagnosis, and management. In conclusion, our findings demonstrate a significant disparity in knowledge about NAFLD among medical specialties, particularly among non‐hepatologists. However, hepatologists have a good understanding of NAFLD, including its diagnostic methods and treatment interventions. On the other hand, non‐hepatologists should exercise caution when examining patients and be able to recognize the disease, promptly referring patients to hepatologists for timely diagnosis. Nonetheless, further studies are needed to explore strategies for increasing knowledge about NAFLD among healthcare professionals, with a particular focus on educating them about the disease.

## Ethics Statement

The study was conducted in line with the market research definition and in accordance with the European Pharmaceutical Market Research Association (EphMRA) and British Healthcare Business Intelligence Association (BHBIA) market research code of conducts. Its only purpose was to capture the opinion of the participants, and no clinical parameter, efficacy, or safety endpoints related to any treatment were investigated. Hence, in line with the guidance provided by the EphMRA, BHBIA, and the National Health Service Health Research Authority (NHS HRA), the research does not qualify as clinical study and Research Ethics Committee review and approval was not required.

## Consent

Participants' consent was collected before participation in the web‐based survey.

## Conflicts of Interest

Laura Colombo is an employee of Viatris.

## Supporting information


Data S1.

## Data Availability

Data sharing is not applicable to this article as no datasets were generated during the current study.
