# Supplementary material for: A Survey Assessing Nonalcoholic Fatty Liver Disease Knowledge Among Hepatologists and Non‐Hepatologists in China
Source: JGH Open. 2024 Dec 10;8(12):e70054. doi: 10.1002/jgh3.70054 (PMC11629256; doi:10.1002/jgh3.70054)
Supplement: Supplementary file 1 — Data S1. [file JGH3-8-e70054-s001.docx]

**Supplementary Table 1** Dietary and lifestyle modifications of NAFLD patients

| **Management of NAFLD** | | | |
| --- | --- | --- | --- |
|  | **Hepatologists** | **Non-hepatologists** | **Overall** |
| Lifestyle modification^*^ | 98.6% | 96.1% | 97.1% |
| Dietary modification^*^ | 98.5% | 95.5% | 96.7% |
| Pharmacological treatment^*^ | 96.4% | 95.3% | 95.7% |
| Bariatric surgery^*^ | 72.2% | 69.8% | 70.7% |
| Other | 24.2% | 24.9% | 24.6% |
| Not sure, would like to receive more information | 0.2% | 2.2% | 1.4% |
| **Dietary management** | | | |
| Low-lipid diet^*^ | 96.7% | 96.4% | 96.5% |
| Low-calories diet^*^ | 92.4% | 88.0% | 89.7% |
| Low-carbohydrate diet^*^ | 86.2% | 83.1% | 84.3% |
| High-protein diet | 62.3% | 51.7% | 55.9% |
| Not sure, would like to receive more information | 0.5% | 2.1% | 1.4% |
| **Lifestyle modification** | | | |
| Avoiding hepatotoxic drugs^*^ | 94.1% | 90.2% | 91.8% |
| Avoiding alcohol^*^ | 91.2% | 87.4% | 89.0% |
| Weight loss 10%^*^ | 71.7% | 66.5% | 68.6% |
| Reducing alcohol use | 41.9% | 42.3% | 42.1% |
| Weight loss of any value | 28.0% | 33.6% | 31.3% |
| Weight loss 3-5%^*^ | 23.7% | 23.0% | 23.3% |
| Not sure, would like to receive more information | 0.3% | 1.8% | 1.2% |
| ^*^Indicate correct answers  NAFLD: non-alcoholic fatty liver disease | | | |

Supplementary Table 2 Drugs worsening NAFLD conditions

| **Drugs that may worsen NAFLD** | | | |
| --- | --- | --- | --- |
| **Types of drugs** | **Hepatologists** | **Non-hepatologists** | **Overall** |
| Paracetamol /acetaminophen | 79.8% | 68.4% | 73.0% |
| MTX | 75.7% | 69.5% | 71.9% |
| Some herbal and food supplements | 68.4% | 57.0% | 61.6% |
| Azathiopirine | 67.5% | 53.7% | 59.2% |
| NSAIDs | 65.7% | 59.6% | 62.1% |
| Some antibiotics | 64.9% | 56.2% | 59.8% |
| Corticosteroids | 60.5% | 49.3% | 53.8% |
| Quinine | 50.6% | 46.7% | 48.3% |
| Statins | 47.7% | 48.7% | 48.3% |
| Tamoxifen | 47.4% | 35.5% | 40.3% |
| Valproate | 44.4% | 37.2% | 40.0% |
| Amiodarone | 41.0% | 41.6% | 41.3% |
| Not sure, would like to receive more information | 2.1% | 6.7% | 4.8% |

NSAID: non-steroidal anti-inflammatory drugs; MTX: methotrexate; NAFLD: non-alcoholic fatty liver disease; NSAIDs: non-steroidal anti-inflammatory drugs

Supplementary Table 3 Ways adopted by HCPs to acquire knowledge about NAFLD and barriers to manage NAFLD patients

| **Usual ways to stay informed about NAFLD** | | | |
| --- | --- | --- | --- |
|  | **Hepatologists** | **Non-hepatologists** | **Overall** |
| Read guidelines and peer-reviewed journals | 97.4% | 86.1% | 90.7% |
| Attend online medical education | 89.5% | 82.1% | 85.1% |
| Attend in person medical education | 64.1% | 52.0% | 56.8% |
| I would like to be more informed | 13.1% | 18.7% | 16.4% |
| HCPs: healthcare professionals; NAFLD: non-alcoholic fatty liver disease | | | |

**Supplementary Table 4** Barriers for management of NAFLD patients

| **Barriers for NAFLD management** | | | |
| --- | --- | --- | --- |
|  | Hepatologists | Non-hepatologists | Overall |
| Lack of compliance by the patient | 94.2% | 89.0% | 91.1% |
| Time constraints | 64.3% | 57.0% | 59.9% |
| Cost of exams | 56.2% | 54.6% | 55.2% |
| Lack of confidence in management | 52.1% | 55.2% | 54.0% |
| Cost of treatment | 50.2% | 50.8% | 50.5% |
| Lack in medical education of doctors | 41.9% | 51.1% | 47.5% |
| Uncomfortable to discuss with patient | 19.6% | 21.1% | 20.4% |
| NAFLD: non-alcoholic fatty liver disease | | | |

**Supplementary Figure 1** Indications of liver biopsy

HCP: healthcare professionals; NAFLD: non-alcoholic fatty liver disease
